# Prenatal maternal and childhood bisphenol a exposure and brain structure and behavior of young children

**DOI:** 10.1186/s12940-019-0528-9

**Published:** 2019-10-15

**Authors:** Melody N. Grohs, Jess E. Reynolds, Jiaying Liu, Jonathan W. Martin, Tyler Pollock, Catherine Lebel, Deborah Dewey, the APrON Study Team, Bonnie J. Kaplan, Catherine J. Field, Deborah Dewey, Rhonda C. Bell, Francois P. Bernier, Marja Cantell, Linda M. Casey, Misha Eliasziw, Anna Farmer, Lisa Gagnon, Gerald F. Giesbrecht, Laksiri Goonewardene, David W. Johnston, Libbe Kooistra, Nicole Letourneau, Donna P. Manca, Jonathan W. Martin, Linda J. McCargar, Maeve O’Beirne, Victor J. Pop, Nalini Singhal

**Affiliations:** 1 Department of Neuroscience, Cumming School of Medicine, University of Calgary, Calgary, Alberta Canada; 2 Owerko Centre, Alberta Children’s Hospital Research Institute, Cumming School of Medicine, University of Calgary, Calgary, Alberta Canada; 3 Department of Radiology, Cumming School of Medicine, University of Calgary, Calgary, Alberta Canada; 4 Department of Laboratory Medicine and Pathology, Faculty of Medicine and Dentistry, University of Alberta, Edmonton, Alberta Canada; 5 Science for Life Laboratory, Department of Environmental Science and Analytical Chemistry, Stockholm University, Stockholm, Sweden; 6 Department of Medical Genetics, Cumming School of Medicine, University of Calgary, Calgary, Alberta Canada; 7 Hotchkiss Brain Institute, Cumming School of Medicine, University of Calgary, Calgary, Alberta Canada; 8 Department of Paediatrics, Cumming School of Medicine, University of Calgary, Calgary, Alberta Canada; 9 Department of Community Health Sciences, Cumming School of Medicine, University of Calgary, Calgary, Alberta Canada; 10 University of Calgary, #397 Owerko Center, Child Development Centre 2500 University Dr. NW, Calgary, Alberta T2N 1N4 Canada

**Keywords:** Magnetic resonance imaging, White matter, Brain development, Bisphenol a, Behavior, Child Behavior Checklist

## Abstract

**Background:**

Bisphenol A (BPA) is commonly used in the manufacture of plastics and epoxy resins. In North America, over 90% of the population has detectable levels of urinary BPA. Human epidemiological studies have reported adverse behavioral outcomes with BPA exposure in children, however, corresponding effects on children’s brain structure have not yet been investigated. The current study examined the association between prenatal maternal and childhood BPA exposure and white matter microstructure in children aged 2 to 5 years, and investigated whether brain structure mediated the association between BPA exposure and child behavior.

**Methods:**

Participants were 98 mother-child pairs who were recruited between January 2009 and December 2012. Total BPA concentrations in spot urine samples obtained from mothers in the second trimester of pregnancy and from children at 3–4 years of age were analyzed. Children participated in a diffusion magnetic resonance imaging (MRI) scan at age 2–5 years (3.7 ± 0.8 years). Associations between prenatal maternal and childhood BPA and children’s fractional anisotropy and mean diffusivity of 10 isolated white matter tracts were investigated, controlling for urinary creatinine, child sex, and age at the time of MRI. Post-hoc analyses examined if alterations in white matter mediated the relationship of BPA and children’s scores on the Child Behavior Checklist (CBCL).

**Results:**

Prenatal maternal urinary BPA was significantly associated with child mean diffusivity in the splenium and right inferior longitudinal fasciculus. Splenium diffusivity mediated the relationship between maternal prenatal BPA levels and children’s internalizing behavior (indirect effect: β = 0.213, CI [0.0167, 0.564]). No significant associations were found between childhood BPA and white matter microstructure.

**Conclusions:**

This study provides preliminary evidence for the neural correlates of BPA exposure in humans. Our findings suggest that prenatal maternal exposure to BPA may lead to alterations in white matter microstructure in preschool aged children, and that such alterations mediate the relationship between early life exposure to BPA and internalizing problems.

## Background

Neurodevelopment is vulnerable to environmental chemical exposure during pregnancy and early childhood [1]. Endocrine-disrupting chemicals (ECDs), including bisphenol A (BPA), are of concern given their presence in food packaging and consumer products (i.e. canned food, personal care products, receipt papers) [2, 3]. Furthermore, there is mounting evidence that early life exposure to EDCs may play a role in the increasing prevalence of neurobehavioral and neurodevelopmental deficits worldwide [1, 4–7]. The World Health Organization (WHO) recommends that increasing global understanding of the effects of chemical exposure during pregnancy and childhood be an international research priority [8].

BPA is a synthetic chemical used in the production of polycarbonate plastics and epoxy resins [2]. Human exposure to BPA is globally ubiquitous [9], with over 90% of North Americans having detectable urinary BPA [3, 10]. Of particular concern is the exposure of pregnant women and the developing fetus [11–13]. BPA can pass through the placenta [14] and cross the blood brain barrier [15], therefore, the developing fetal brain is likely exposed throughout gestation [11]. Specifically, following exposure, BPA is rapidly metabolized into various conjugates [16] (i.e., glucuronides and sulphates) to allow for efficient renal clearance. However, a small portion of free, estrogenically-active BPA can avoid first-pass metabolism and circulate throughout the body [17–19]. BPA conjugates may linger in the fetoplacental compartment and become reactivated through deconjugation within fetal tissue, leading to greater fetal exposure [20, 21]. Biomonitoring studies have also demonstrated continued exposure postnatally, with BPA being detected within urine samples of children [7, 22]. Therefore, there is ample evidence supporting that fetuses, newborn infants, and children are exposed to BPA throughout development.

Exposure to BPA may interfere with the organization of neurochemical and neuroendocrine systems, which could alter typical neurodevelopment and behavior [7, 23–29]. Previous research has shown that prenatal exposure may program later brain development [30–32]. Prenatal exposure to BPA in rodents, at levels below the oral reference dose (maximum acceptable oral intake of a toxic substance) of 50 μg/kg/day set by the U.S. EPA [33], has been associated with long-lasting neurodevelopmental changes, including increased anxiety [25, 34], cognitive deficits [25, 34–36], and behavioral alterations [37–43].

Animal studies also suggest altered brain structure and development with perinatal exposure to BPA [24]. For instance, inhibited oligodendrocyte cell differentiation and de-compacted myelin have been noted with early exposure to BPA [44]. Behavioral findings from animal studies are consistent with human epidemiological studies, which report associations between BPA exposure and adverse behavioral outcomes, such as increased internalizing (i.e., anxiety, depression) and externalizing (i.e., attention) problems in children [4, 5, 7, 45–54]. There are clear behavioral consequences of BPA exposure; however, the underlying effects on human brain structure are unclear. Rodent studies show that white matter is particularly sensitive to early exposure to BPA [44], suggesting that it is an important avenue to investigate in order to better understand the effects of BPA in humans.

Diffusion tensor imaging (DTI) is a safe and non-invasive neuroimaging technique that is sensitive to brain white matter microstructure. Given the limited understanding of the neural correlates for BPA exposure in humans, the current study, utilizing DTI, investigated the associations between prenatal maternal and childhood BPA exposure and white matter microstructure in children. We examined these relationships in young children (2 to 5 years), as early childhood is a critical developmental period where considerable white matter development takes place [55]. Additionally, we assessed both prenatal maternal and early childhood BPA exposure to better understand if differences in the time of exposure are related to brain structure. Previous studies have shown that widespread alterations in white matter microstructure are associated with higher scores on internalizing (i.e., anxiety and depression) [56, 57] and externalizing (i.e., attention) [58, 59] behaviors measured using the Child Behavior Checklist (CBCL) [60]. Therefore, we also investigated if altered behavior associated with early life exposure to BPA was related to underling alterations in white matter microstructure. To accomplish this, post-hoc analyses were conducted to investigate whether white matter alterations mediated the associations between BPA exposure and behavior that have been commonly reported in pediatric studies.

## Methods

### Participants

Participants were recruited from an ongoing prospective birth cohort, the Alberta Pregnancy Outcomes and Nutrition (APrON) study. APrON is a longitudinal study following women and their offspring (*n* = 2189 mothers, *n* = 2169 children) [61]. Women were initially recruited during pregnancy between January 2009 to December 2012. Women provided informed consent and completed bio-sampling as well as comprehensive questionnaires. Their children were assessed for this study between December 2013 to December 2016. Children completed bio-sampling, comprehensive neuropsychological testing and a subset also completed magnetic resonance imaging (MRI) scans. Parents provided consent regarding their children’s participation, and children were monitored through regular follow-ups on comfort and state before, during and after data collection.

Mother-child pairs for the current study were selected based on the presence of a maternal (2nd trimester) urine sample and successful child neuroimaging data (*n* = 98). At the time of the MRI, children ranged in age from 2.47 to 5.99 years (mean age: 3.7 ± 0.8, *n* = 50 female, *n* = 48 male). Gestational age at birth ranged from 36 to 41 weeks (mean gestation age: 39 ± 1.26). All children were healthy and free of diagnosed neurological or neurodevelopmental disorders. Of these 98 children, 77 had a urine sample collected between 3.0–4.8 years and 56 had the CBCL completed by their parents within 6 months of the MRI. This study was approved by the University of Calgary Health Research Ethics Board (REB14–1702; REB13–0020).

### Urine sample collection and analysis

Total BPA concentrations were analyzed in spot maternal urine samples collected during the 2nd trimester of pregnancy (*n* = 98, mean gestation: 17 ± 2.1 weeks) and in child spot urine samples collected at 3–4 years of age (*n* = 77, mean age: 4.2 ± 0.5 years). Detailed information of the collection and analytical methods have been reported previously [27, 62]. In brief, urine was collected in sterile urine cups and was immediately aliquoted into 9 mL cryovials then stored at − 80’C. Quantitative analysis involved deconjugation using a mixture of β-glucuronidase and sulfatase. Total BPA concentrations were then quantified using online solid-phase extraction coupled to liquid chromatography-Orbitrap mass spectrometry (Orbitrap Elite, Thermo, Fisher Scientific, San Jose, CA). Isotope-labeled BPA (bisphenol-A-(diphenyl-13C12)) was used as internal standard for quantification. Recovery of BPA spiked at two levels (2.5 ng/mL and 12.5 ng/mL) ranged from 95 to 116%, with a relative standard deviation (RSD) of 3–12%. Calibration linearity was valid between 0.5 ng/mL and 50 ng/mL, and regression coefficients of standard curves (6-point curve) were always > 0.99. Urine samples with concentrations above 50 ng/mL were diluted and injected again for accurate quantification within the range of calibration. The limit of detection (LOD) for total BPA in spot urine samples was 0.32 ng/mL. Total BPA concentrations below the LOD were substituted by *LOD*/ √ 2 for statistical analyses. Finally, potential contamination of target analytes due to sampling and storage was tested using pure water as a surrogate for urine at the time of sample collection, storage and analysis.

Aliquots from the same urine samples were also analyzed for creatinine in the Clinical Trials Laboratory, Alberta Health Services (Edmonton, Alberta), to control for urinary dilution in our models. The LOD of creatinine was 10 mg/dL and the inter-day relative standard deviation of duplicate injections of a reference urine sample over 4 months was 2% (*n* = 48 reference urine samples sent for creatinine detection).

### MRI acquisition

All imaging took place at the Alberta Children Hospital (ACH) on a General Electric 3 T MR750w MRI scanner, using a 32-channel head coil (GE, Waukesha, WI). Children were scanned while watching a movie or naturally asleep; for further details on MRI preparation methods see Thieba and colleagues (2018) [63]. Whole-brain diffusion weighted images were acquired using a single shot spin echo echo-planar imaging protocol, with 30 diffusion encoding gradient directions (b = 750 s/mm^2^) and 5 non-diffusion-weighted directions (b = 0 s/mm^2^), TR = 6750 ms, TE = 79 ms, spatial resolution of 1.6 × 1.6 × 2.2 mm^3^ resampled to 0.78 × 0.78 × 2.2 mm^3^ on the scanner, total sequence time = 4:03 min.

### DTI processing

DTI data was visually inspected prior to processing. Detection and removal of motion-corrupted volumes was performed manually by an investigator blinded to participant demographics. All participants had at least 18 low-motion diffusion-weighted and 3 low-motion non-diffusion-weighted volumes remaining (mean number of total volumes: 31 ± 3.6). Data was then processed through ExploreDTI (V4.8.6) [64] for signal drift, Gibbs free ringing, eddy currents, and motion corrections.

**Fig. 1:**
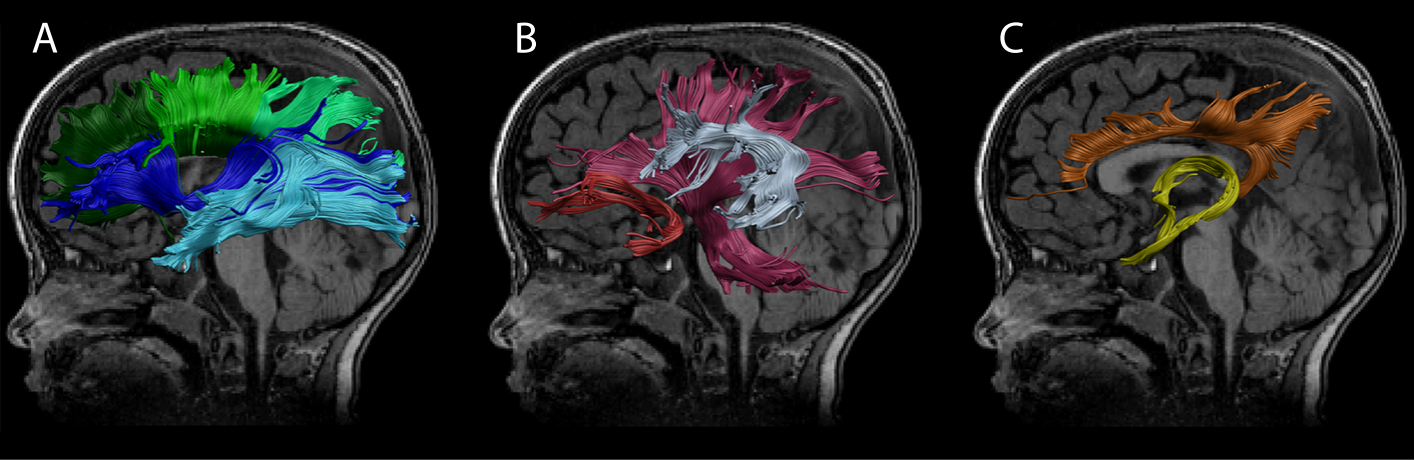
Isolated white matter tracts. Diffusion measures were calculated for 10 unique white matter tracts; **a**) dark green: genu of corpus callosum, lime green: body of corpus callosum, mint green: splenium of corpus callosum, dark blue: left inferior fronto-occipital fasciculus (IFO), light blue: left inferior longitudinal fasciculus (ILF); **b**) pink: left pyramidal, silver: left superior longitudinal fasciculus including the arcuate fasciculus (SLF), red: left uncinate fasciculus (UF); **c**) orange: left cingulum bundle, yellow: fornix. Tracts are shown on a T1-weighted image from a male 3.7 years of age

Deterministic semi-automated tractography was performed in ExploreDTI, using a priori information of tract location [65] for 10 major white matter tracts: the genu, body, and splenium of the corpus callosum, the fornix, and the left and right hemisphere tracts for the pyramidal fibers, cingulum, inferior fronto-occipital fasciculus (IFO), inferior longitudinal fasciculus (ILF), superior longitudinal fasciculus including arcuate (SLF), and uncinate fasciculus (UF) (Fig. 1; region of interest (ROI) placements for tract extraction [66]). All generated tracts were visualized and manually corrected if necessary. Averaged values of fractional anisotropy (FA) and mean diffusivity (MD) were extracted for each tract (left and right separately), resulting in 32 diffusion measurements (FA and MD for 16 total white matter tracts). FA and MD provide indirect measures of white matter tract microstructure and are sensitive to microscopic features such as various axonal properties (i.e., myelination, diameter, density, coherence) [67]. For instance, increased FA and decreased MD may reflect more coherent fibers, increased myelination and/or increased fiber density [68, 69].

### Behavioral outcomes

Of the 98 children who underwent MRI, 56 had the CBCL [60] completed by their parents within 6 months of the MRI. The CBCL is a parent report that provides a reliable measure [70] of children’s internalizing (i.e., anxious, depressive) and externalizing (i.e., aggressive, hyperactive, noncompliant) behaviors and has been utilized in various studies examining the relationship between BPA and child behavior [71, 72]. Higher scores on this measure indicate more behavioral problems. Lastly, internalizing and externalizing behaviors are predictors of later disorders (i.e., depression) as well as a heightened risk for substance use and criminal behavior [73, 74].

### Statistical analysis

Statistical analyses were conducted in SPSS (IBM Statistics V22.0.0, https://www.software.ibm.com). Prenatal maternal and childhood total BPA and urinary creatinine concentrations were natural log transformed.

#### BPA exposure and childhood brain structure

The primary analyses included a series of linear regressions that examined the relationship between BPA concentrations and FA and MD, separately for each tract and time point (prenatal maternal or childhood). Covariates included child sex and age at scan, as well as urinary creatinine levels (maternal or child, as relevant). A follow-up prenatal maternal BPA analysis was conducted, controlling for childhood BPA levels, with all other covariates remaining the same as the model above. Additionally, child sex and age were tested as moderators to examine potential interaction effects using PROCESS75 (V2.16.3). We report results uncorrected and corrected for multiple comparisons using false discovery rate (FDR).

#### BPA, brain structure and behavior

Linear regressions examined the relationship between CBCL internalizing and externalizing behavior scores and BPA, as well as between CBCL scores and white matter tracts identified as being significantly related to BPA exposure in the primary analysis above. Covariates included child sex and age at scan, as well as maternal creatinine and child BPA levels (as relevant).

#### Mediation analyses

Based on the results of the above regressions, additional post-hoc analyses were run to investigate whether alterations in white matter microstructure mediated the relationship between BPA exposure and internalizing and externalizing behaviour problems. Within PROCESS (V2.16.3) [75], a mediation analysis (using the bootstrapping method to test significance of total and indirect effects) was conducted, with BPA as the predictor (X), CBCL score as the outcome (Y), tract diffusion measure as the mediator, and creatinine, child sex, and age at scan as covariates; the bootstrapping method is recommended for small sample sizes with temporal distance between the independent and dependent variables [76–79].

## Results

Participant age and sex, as well as mean BPA and CBCL T-scores are shown in Table 1. Child sex, CBCL Internalizing and CBCL Externalizing scores, as well as average maternal prenatal and child BPA were not significantly different in the subsample of participants (*n* = 56), with both an MRI and completed CBCL compared to the all of the children with MRI (*n* = 98). However, children in the subsample were significantly older (*n* = 56) (*p* = 0.003).

**Table 1:** Characteristics of children with MRI compared to those with both MRI and completed CBCLs

Demographic(s)	MRI (*n* = 98) M (SD)	MRI and CBCL (*n* = 56) M (SD)	*p-*value
Child Sex (% female)	51	50	0.880
Child Age at MRI (years)	3.73 (0.84)	4.09 (0.84)	**0.003**
Maternal BPA	2.59 (4.39)	2.62 (3.47)	0.947
Childhood BPA	1.65 (1.94)	1.59 (2.05)	0.836
CBCL Internalizing problems^a^	–	47.57 (9.72)	–
CBCL Externalizing problems^a^	–	47.86 (10.02)	–

*MRI* Magnetic resonance imaging, *CBCL* Child Behavior Checklist

^a^CBCL T Score

Child CBCL Internalizing T-Scores ranged from 29 to 78, with a mean of 47.57 ± 9.71; Externalizing T-Scores ranged from 28 to 67, with a mean of 47.86 ± 10.02. These mean T-scores were in the normal range for this standardized measure (M = 50; SD = 10).

BPA was detectable in 89% of maternal urine samples (Geometric Mean (GM) 1.4 ng/mL) and 91% of childhood urine samples (GM 1.0 ng/mL). Average BPA concentrations (Table 2) were similar to those reported in Canadian national biomonitoring data (i.e., Canadian Health Measures Survey: 1.1 μg/mL, ranging from 0.9–1.4 μg/mL for adults aged 20–39 years; 1.2 μg/mL, ranging from 1.0–1.4 μg/mL for children aged 3–5 years) [3, 80]. In the same spot urine samples, average maternal creatinine levels were 103.3 ± 645 mg/dL, whereas average child creatinine levels were 64.1 ± 266 mg/dL.

**Table 2:** Total BPA geometric means, percentiles, and maximum concentrations in maternal and child urine samples

	Detection Frequency (% > LOD)	Urinary Total BPA (ng/mL)					
		GM (95% CI)	P25%	P50%	P75%	P95%	Max
Maternal (*n* = 98)^a^	88.8	1.4 (0.5, 2.2)	0.5	1.5	2.7	10.7	34.9
Child (*n*= 77) ^b^	91.0	1.0 (0.6, 1.5)	0.5	0.9	1.8	6.1	10.9

*LOD* limit of detection, *GM* geometric mean, 95% CI: confidence interval of GM, P25: 25th percentile; P50: 50th percentile; P75: 75th percentile; P95: 95th percentile. MAX: maximum concentrations

^a^collected in the second trimester of pregnancy

^b^child urine samples collected at 3–4 years of age; 21 children did not complete the 3–4 year urine sampling

In models adjusted for child sex and child age at scan, and maternal urinary creatinine, prenatal maternal urinary BPA concentrations were significantly associated with MD of the splenium (*p* = 0.046, *β* = 0.238, [CI: 0.005 0.471]) and the right inferior longitudinal fasciculus (*p* = 0.017, *β* = 0.249, [CI: 0.046, 0.452]). When controlling for childhood urinary BPA, associations remained significant (*p* < 0.05). MD and FA of all remaining white matter pathways were not significantly related to prenatal maternal BPA concentrations (*p* > 0.05) (see Table 3 for regression coefficients). Results did not withstand FDR correction for multiple comparisons.

**Table 3:** Associations between prenatal maternal urinary BPA and white matter tract parameters by child sex

Tract	DTI Parameter	All Children *B (95% CI)* *(N = 98)*	Boys *B (95% CI)* *(N = 48)*	Girls *B (95% CI)* *(N = 50)*	Tract x Sex *p*-value
Left Cingulum	FA	0.007 (− 0.213, 0.226)	0.016 (− 0.240, 0.266)	− 0.031 (− 0.435, 0.360)	0.738
	MD	0.056 (− 0.156, 0.269)	0.087 (− 0.203, 0.367)	− 0.022 (− 0.367, 0.323)	0.780
Right Cingulum	FA	0.133 (− 0.089, 0.354)	0.194 (− 0.123, 0.470)	0.077 (− 0.250, 0.427)	0.407
	MD	0.090 (− 0.116, 0.295)	0.119 (− 0.163, 0.372)	0.026 (− 0.313, 0.369)	0.646
CC Body	FA	0.034 (− 0.195, 0.262)	− 0.146 (− 0.390, 0.140)	0.286 (− 0.063, 0.740)	0.434
	MD	0.041 (− 0.172, 0.254)	0.043 (− 0.258, 0.340)	− 0.001 (− 0.326, 0.325)	0.539
CC Genu	FA	0.051 (− 0.174, 0.276)	− 0.076 (− 0.322, 0.199)	0.191 (− 0.182, 0.639)	0.289
	MD	− 0.102 (− 0.320, 0.116)	− 0.077 (− 0.335, 0.196)	− 0.115 (− 0.519, 0.255)	0.505
CC Splenium	FA	0.035 (− 0.209, 0.280)	− 0.135 (− 0.433, 0.182)	0.302 (− 0.071, 0.743)	0.380
	MD	0.238 (0.005, 0.471)*	0.140 (− 0.173, 0.430)	0.355 (− 0.007, 0.771)	0.169
Fornix	FA	− 0.016 (− 0.259, 0.227)	0.082 (− 0.242, 0.405)	− 0.157 (− 0.558, 0.228)	0.383
	MD	0.030 (− 0.203, 0.263)	0.039 (− 0.296, 0.377)	0.022 (− 0.330, 0.372)	0.802
Left IFO	FA	0.169 (− 0.035, 0.373)	0.190 (− 0.102, 0.460)	0.154 (− 0.153, 0.494)	0.691
	MD	− 0.040 (− 0.239, 0.159)	0.010 (− 0.253, 0.271)	− 0.151 (− 0.487, 0.161)	0.872
Right IFO	FA	− 0.080 (− 0.302, 0.142)	−0.042 (− 0.274, 0.208)	−0.095 (− 0.539, 0.303)	0.316
	MD	0.182 (−0.037, 0.401)	0.280 (−0.011, 0.566)	− 0.020 (− 0.369, 0.328)	0.769
Left ILF	FA	0.008 (−0.200, 0.217)	0.084 (− 0.198, 0.358)	−0.100 (− 0.445, 0.238)	0.467
	MD	0.072 (−0.116, 0.261)	0.133 (−0.157, 0.402)	− 0.026 (− 0.294, 0.245)	0.777
Right ILF	FA	−0.146 (− 0.378, 0.086)	−0.106 (− 0.435, 0.222)	−0.199 (− 0.565, 0.153)	0.687
	MD	0.249 (0.046, 0.452)*	0.352 (0.067, 0.673)*	0.011 (−0.259, 0.277)	0.566
Left Pyramidal	FA	0.017 (−0.210, 0.244)	−0.038 (− 0.279, 0.217)	0.112 (− 0.292, 0.563)	0.926
	MD	0.066 (−0.145, 0.278)	0.045 (− 0.263, 0.355)	0.069 (− 0.242, 0.364)	0.487
Right Pyramidal	FA	0.006 (−0.216, 0.227)	−0.104 (− 0.431, 0.216)	0.217 (− 0.120, 0.518)	0.340
	MD	0.180 (−0.043, 0.403)	0.270 (− 0.040, 0.640)	−0.040 (− 0.326, 0.259)	0.368
Left SLF	FA	0.002 (− 0.221, 0.226)	−0.025 (− 0.301, 0.258)	0.033 (− 0.349, 0.428)	0.860
	MD	−0.005 (− 0.210, 0.199)	0.032 (− 0.241, 0.299)	− 0.085 (− 0.421, 0.254)	0.948
Right SLF	FA	− 0.007 (− 0.223, 0.209)	−0.009 (− 0.285, 0.268)	0.043 (− 0.310, 0.406)	0.435
	MD	0.080 (−0.118, 0.279)	0.095 (− 0.159, 0.332)	− 0.005 (− 0.332, 0.322)	0.276
Left Uncinate	FA	0.091 (− 0.135, 0.316)	0.029 (− 0.274, 0.328)	0.237 (− 0.082, 0.603)	0.671
	MD	−0.025 (− 0.239, 0.190)	0.208 (− 0.066, 0.490)	− 0.404 (− 0.726, − 0.080)*	**0.010**
Right Uncinate	FA	− 0.030 (− 0.253, 0.193)	− 0.148 (− 0.404, 0.144)	0.144 (− 0.219, 0.551)	0.685
	MD	−0.002 (− 0.231, 0.227)	−0.505 (− 0.774,-0.246)	−0.096 (− 0.532, 0.308)	0.229

Note: *p <.05

*FA* fractional anisotropy, *MD* mean diffusivity, *CC* corpus callosum, *IFO* inferior fronto-occipital fasciculus, *ILF* inferior longitudinal fasciculus, *SLF* superior longitudinal fasciculus

Models including all children were adjusted for maternal urinary creatinine, child sex and child age at scan. Models stratified by sex were adjusted for maternal urinary creatinine and child age at scan

A significant interaction of child sex with prenatal X maternal urinary BPA for left uncinate MD was observed (*p =* 0.01); no other sex interaction effects were noted. A significant interaction of child age and prenatal maternal urinary BPA was observed for fornix FA (*p =* 0.04). Age did not significantly modify any other associations between white matter tract parameters and maternal urinary BPA levels (see Tables 3 and 4 for results of regression models, and models stratified by child sex or median age).

**Table 4:** Associations between prenatal maternal urinary BPA and white matter tract parameters by child age

Tract	DTI Parameter	All Children *B (95% CI)* *(N = 98)*	Younger (< 3.52 yrs) *B (95% CI)* *(N = 49)*	Older (> 3.52 yrs) *B (95% CI)* *(N = 49)*	Tract x Age *p*-value
Left Cingulum	FA	0.007 (− 0.213, 0.226)	− 0.183 (− 0.506, 0.156)	0.058 (− 0.262, 0.373)	0.716
	MD	0.056 (− 0.156, 0.269)	−0.055 (− 0.414, 0.310)	0.151 (− 0.150, 0.444)	0.260
Right Cingulum	FA	0.133 (−0.089, 0.354)	0.013 (− 0.312, 0.333)	0.155 (− 0.162, 0.488)	0.775
	MD	0.090 (−0.116, 0.295)	−0.096 (− 0.441, 0.265)	0.258 (− 0.031, 0.551)	0.115
CC Body	FA	0.034 (−0.195, 0.262)	0.013 (− 0.392, 0.418)	0.060 (− 0.234, 0.334)	0.596
	MD	0.041 (− 0.172, 0.254)	−0.024 (− 0.382, 0.336)	0.094 (− 0.207, 0.388)	0.278
CC Genu	FA	0.051 (−0.174, 0.276)	0.198 (− 0.176, 0.558)	−0.057 (− 0.385, 0.273)	0.682
	MD	−0.102 (− 0.320, 0.116)	−0.295 (− 0.659, 0.090)	0.039 (− 0.274, 0.345)	0.330
CC Splenium	FA	0.035 (−0.209, 0.280)	0.151 (− 0.231, 0.531)	0.024 (− 0.311, 0.360)	0.972
	MD	0.238 (0.005, 0.471)*	0.045 (−0.290, 0.372)	0.293 (−0.026, 0.670)	0.648
Fornix	FA	−0.016 (− 0.259, 0.227)	−0.348 (− 0.726, 0.030)	0.335 (0.010, 0.661)*	**0.040**
	MD	0.030 (−0.203, 0.263)	−0.057 (− 0.403, 0.297)	0.061 (− 0.291, 0.422)	0.438
Left IFO	FA	0.169 (−0.035, 0.373)	0.090 (− 0.224, 0.356)	0.193 (− 0.162, 0.607)	0.575
	MD	−0.040 (− 0.239, 0.159)	−0.105 (− 0.446, 0.252)	−0.058 (− 0.355, 0.244)	0.852
Right IFO	FA	−0.080 (− 0.302, 0.142)	−0.299 (− 0.515, 0.065)	0.046 (− 0.347, 0.456)	0.928
	MD	0.182 (−0.037, 0.401)	0.215 (− 0.154, 0.581)	0.103 (− 0.212, 0.402)	0.878
Left ILF	FA	0.008 (−0.200, 0.217)	0.207 (−0.162, 0.564)	− 0.115 (− 0.415, 0.199)	0.628
	MD	0.072 (−0.116, 0.261)	0.076 (− 0.261, 0.396)	0.103 (− 0.200, 0.413)	0.314
Right ILF	FA	−0.146 (− 0.378, 0.086)	−0.268 (− 0.675, 0.122)	0.019 (− 0.285, 0.318)	0.327
	MD	0.249 (0.046, 0.452)*	0.284 (−0.089, 0.645)	0.240 (−0.050, 0.480)	0.969
Left Pyramidal	FA	0.017 (−0.210, 0.244)	−0.074 (− 0.384, 0.261)	0.078 (− 0.298, 0.477)	0.567
	MD	0.066 (−0.145, 0.278)	− 0.095 (− 0.422, 0.248)	0.152 (− 0.167, 0.484)	0.289
Right Pyramidal	FA	0.006 (− 0.216, 0.227)	−0.080 (− 0.392, 0.255)	0.095 (− 0.257, 0.469)	0.496
	MD	0.180 (−0.043, 0.403)	0.109 (− 0.240, 0.429)	0.223 (− 0.114, 0.608)	0.221
Left SLF	FA	0.002 (−0.221, 0.226)	−0.248 (− 0.569, 0.126)	0.128 (− 0.212, 0.485)	0.746
	MD	−0.005 (− 0.210, 0.199)	−0.058 (− 0.405, 0.296)	0.028 (− 0.274, 0.330)	0.223
Right SLF	FA	−0.007 (− 0.223, 0.209)	−0.218 (− 0.535, 0.152)	0.131 (− 0.185, 0.459)	0.642
	MD	0.080 (−0.118, 0.279)	0.041 (− 0.291, 0.362)	0.093 (− 0.206, 0.394)	0.242
Left Uncinate	FA	0.091 (−0.135, 0.316)	0.045 (−0.290, 0.363)	0.126 (− 0.230, 0.517)	0.260
	MD	−0.025 (− 0.239, 0.190)	−0.166 (− 0.498, 0.205)	0.054 (− 0.282, 0.390)	0.304
Right Uncinate	FA	−0.030 (− 0.253, 0.193)	0.004 (− 0.354, 0.362)	−0.004 (− 0.341, 0.332)	0.451
	MD	−0.002 (− 0.231, 0.227)	0.064 (− 0.304, − 0.422)	−0.113 (− 0.449, 0.221)	0.320

Note: *p < .05

*FA* fractional anisotropy, *MD* mean diffusivity, *CC* corpus callosum, *IFO* inferior fronto-occipital fasciculus, *ILF* inferior longitudinal fasciculus, *SLF* superior longitudinal fasciculus

Models including all children were adjusted for maternal urinary creatinine, child sex and child age at scan. Models stratified by median age were adjusted for maternal urinary creatinine and child sex

Post-hoc regressions adjusting for child sex and age at scan, maternal urinary creatinine, and child urinary BPA levels, showed a significant relationship between prenatal maternal BPA concentrations and internalizing (*p* = 0.009, *β* = 0.356, [CI: 0.095, 0.652]), but not externalizing (*p* > 0.1) CBCL scores (see Table 5 for results of unadjusted and adjusted models). FA and MD of the splenium were significantly associated with both internalizing (FA: *p* = 0.011, *β* = − 0.345, [CI: − 0.601, − 0.082]; MD: *p* = 0.010, *β* = 0.356, [CI: 0.087, 0.599]) and externalizing (FA: *p* = 0.006, *β* = − 0.365, [CI: − 0.617, − 0.107]; MD: *p* = 0.006, *β* = 0.377, [CI: 0.111, 0.615]) CBCL scores. In contrast, FA of the right inferior longitudinal fasciculus was significantly associated to externalizing (*p* = 0.032, *β* = − 0.300, [CI: − 0.569, − 0.027]), but not internalizing (*p* > 0.1) CBCL scores; MD of the right inferior longitudinal fasciculus was not significantly associated to internalizing or externalizing behavior (*p* > 0.1*)* (see Table 6 for results of unadjusted and adjusted models).

**Table 5:** Association between prenatal maternal urinary BPA and child CBCL scores for internalizing and externalizing behaviors

Maternal Urinary BPA	All Children *B (95% CI)* *Unadjusted*	All Children *B (95% CI)* *Adjusted*
CBCL Internalizing	0.330 (0.056, 0.638)*	0.356 (0.095, 0.652)*
CBCL Externalizing	0.148 (− 0.152, 0.467)	0.229 (− 0.108, 0.589)

Note: *p < .05

Unadjusted models and models adjusted for maternal creatinine, child urinary BPA levels, child sex and age at scan, are shown

**Table 6:** Association between CBCL scores and white matter tract parameters for tracts identified as associated with prenatal maternal urinary BPA levels

	DTI Parameter	All Children *B (95% CI)* *Unadjusted*	All Children *B (95% CI)* *Adjusted*
CBCL Internalizing			
CC Splenium	FA	−0.352 (− 0.602, − 0.096)*	−0.345 (− 0.601, − 0.082)*
	MD	0.362 (0.104, 0.594)*	0.356 (0.087, 0.599)*
Right ILF	FA	−0.141 (− 0.408, 0.128)	−0.132 (− 0.413, 0.152)
	MD	0.128 (−0.133, 0.371)	0.104 (− 0.209, 0.401)
CBCL Externalizing			
CC Splenium	FA	−0.350 (− 0.600, − 0.093)*	−0.365 (− 0.617, − 0.107)*
	MD	0.342 (0.082, 0.576)*	0.377 (0.111, 0.615)*
Right ILF	FA	−0.241 (− 0.501, 0.024)	−0.300 (− 0.569, − 0.027)*
	MD	0.116 (− 0.144, 0.360)	0.232 (− 0.083, 0.514)

Note: *p < .05

*FA* fractional anisotropy, *MD* mean diffusivity, *CC* corpus callosum, *ILF* inferior longitudinal fasciculus

Unadjusted and models adjusted for child sex and age at scan, are shown

**Fig. 2:**
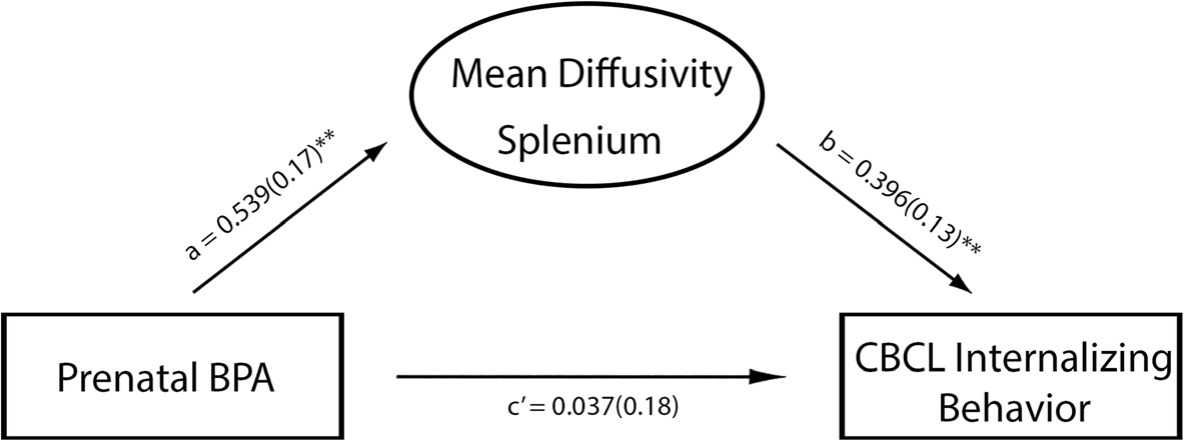
Mediation model showing associations between prenatal BPA levels, child white matter microstructure of splenium and child internalizing behavior. Standardized beta coefficients, and standard errors in brackets, are reported; **p ≤ 0.01. Note that the final model was adjusted for child sex and age at scan, as well as maternal urinary creatinine

A mediation model adjusted for child sex and age at scan, as well as maternal urinary creatinine showed a significant total effect for prenatal maternal BPA and splenium MD (path a: *p* = 0.003, β = 0.539, [CI: 0.190, 0.888]), as well as splenium MD and CBCL internalizing behavior (path b: *p* = 0.004, β = 0.396, [CI: 0.134, 0.657]). The direct effect of prenatal maternal BPA and child internalizing behavior was not significant (path c’: *p* = 0.836, β = 0.037, [CI: − 0.317, 0.391]). Finally, splenium MD significantly mediated the relationship of prenatal maternal BPA and internalizing behavior, as demonstrated by a significant indirect effect (path ab: β = 0.213, [CI: 0.017, 0.564]) (Fig. 2).

No significant associations were observed between childhood urinary BPA concentrations and FA or MD for any of the isolated white matter tracts (*p* > 0.05).

## Discussion

In this first human study of BPA exposure and brain structure, higher prenatal maternal BPA levels were related to higher diffusivity in the splenium and right inferior-longitudinal fasciculus of young children. These relationships suggest that children who were prenatally exposed to higher doses of BPA during the second trimester of pregnancy may have less developed white matter in inferior and posterior brain regions compared to children exposed to lower doses of BPA.

Animal studies show that perinatal BPA exposure alters brain structure and development [24], inducing precocious neurogenesis [81], altering synaptic transmission and arrangement [36, 82], and inhibiting oligodendrocyte cell differentiation and de-compacted myelin [44]. Animal studies also report negative effects of BPA on behavior, including increased anxiety and deficits in learning, memory, and social behavior [83, 84]. Although inconsistent, similar behavioral outcomes have been associated with prenatal maternal BPA exposure in human studies [7]. Here, we show evidence of altered white matter microstructure in young children exposed to greater BPA levels prenatally.

Anisotropy (i.e., FA) and diffusivity (i.e., MD) are related to microscopic (i.e., axonal myelination, density, and diameter) and macroscopic features (i.e., consistency of axonal direction and volume of white matter within a voxel) of brain white matter [67]. Our findings of higher MD within the splenium and right inferior longitudinal fasciculus in children exposed to higher prenatal maternal BPA, are likely related to decreased axon packing, myelination, and/or fiber coherence. FA increases and MD decreases with age in typically developing children [85], so higher MD may suggest that white matter is less developed in children who experienced higher prenatal maternal BPA exposure. Significant changes of diffusivity without accompanying changes of anisotropy, may indicate that structural characteristics (i.e., fiber density) affected by prenatal maternal BPA exposure are altering overall water movement, while preserving anisotropy [86].

The inferior longitudinal fasciculus has been associated with anxiety, depression and attention [87–89]. Additionally, both splenium and inferior longitudinal fasciculus white matter microstructure are associated with visual processing skills [90, 91]. Previous research has noted deficits in executive functioning [5], as well as increased internalizing (i.e., anxiety, depression) and externalizing (i.e., attention) behavior in children exposed to BPA prenatally [7, 92, 93]. Findings from the current study, specifically the mediation analysis, provides support for the hypothesis that underlying alterations in white matter microstructure may be a mechanism by which early life exposure to BPA induces negative behavioral outcomes.

We found limited evidence of sex dependent effects on the relationships between BPA exposure and brain white matter microstructure. Sex differences in the prevalence of internalizing and externalizing problems have been demonstrated in studies using the CBCL [94]. Additionally, previous research has noted inconsistent findings regarding the influence of sex on the relationship between BPA exposure and behavioral outcomes in children [7]. The fact that few sex effects were noted in the present study may suggest that maternal prenatal exposure to BPA affects white matter microstructure similarly in both female and male children at this young age. It is possible that sex differences in brain structure may emerge later in development, as internalizing problems worsen [94].

Brain development begins early in gestation, with myelination beginning in the second trimester [95, 96]. White matter development and myelination continue into young adulthood [96–98]. This rapid development may leave the brain vulnerable to both prenatal and postnatal neurotoxicant exposure [99, 100]. Our findings of associations between prenatal maternal BPA levels and white matter structure, but the lack of similar findings with childhood exposure, suggest that the prenatal period is more sensitive to BPA exposure.

Animal literature has explored the molecular mechanisms by which BPA may alter white matter structure, demonstrating that BPA disrupts gene expression during development [84, 101]. Early exposure to BPA dysregulates gene expression that is critical to the proliferation and differentiation of oligodendrocyte progenitor cells, and it decreases the expression of genes and proteins critical to myelination [81]. White matter development during the second and third trimesters is highly dependent on intrinsic (i.e., transcriptional regulation) and extrinsic (i.e., extracellular ligands, secreted molecules) signaling mechanisms [102]. Therefore, exposure to BPA during this time may alter the establishment (i.e., bundle fasciculation), organization (i.e., pre-myelination) [103], and myelination of white matter through epigenetic modifications of early gene expression. Such alterations may prenatally program the fetus for different trajectories of later brain development [104–106].

Although animal studies have consistently reported findings regarding the disrupting effects of BPA exposure on white matter structure and development (i.e., altered neurogenesis, transmission, myelination etc.), the limited associations between BPA and white matter microstructure observed in the present study may be due, at least in part, to differences between human exposure levels and exposure levels noted within the animal literature. Average human BPA intake is estimated to be 40–80 ng/kg/day, based on national biomonitoring data from Canada [3] and the United States [80]. BPA levels in the current study were similar to Canadian national biomonitoring data [3, 80], with few participants above average exposure levels. Animal studies often employ a wide range of doses that are not necessarily equivalent to human exposures. Thus, it is likely that the effects of BPA on humans are subtler than those observed in animals at very high doses. Imaging studies, which oversample children at the high end of BPA exposure levels, may be able to better elucidate the relationship of BPA exposure and brain structure.

This study has several limitations. Human exposure and excretion patterns show temporal variability, and in the current study, BPA concentrations were taken from a single spot urine sample. Although previous literature has supported moderate sensitivity of single-spot sample collection [107] and suggests that single-spot sampling may adequately reflect average exposure of the population [108], recent literature encourages repeated biospecimen collection [109]. Future studies monitoring for cumulative BPA exposure with more frequent measurements (i.e., during each gestational period and postnatal year) and with multiple specimens per participant, will be able to better determine relationships between BPA exposure and white matter throughout different stages of development. The two significant results seen here did not survive multiple comparison correction. It may be that we are underpowered due to a small sample size or that this relationship may be more clearly observed with higher exposures. Alternatively, findings may reflect a skewed sample and/or other confounding factors. Therefore, future studies are necessary to determine the validity of these findings. Finally, given that childhood is a period of rapid structural brain development [110, 111], longitudinal studies will be critical for determining whether prenatal and/or postnatal BPA exposure are associated with altered trajectories of brain maturation.

## Conclusions

Here, in the first human study of brain structure and early-life BPA exposure, higher maternal BPA concentrations during the second trimester of pregnancy were associated with microstructural alterations in inferior and posterior white matter tracts of young children. Further, these white matter alterations mediated the relationship between prenatal BPA and child internalizing behavior problems. Therefore, our results suggest that alterations to brain structure may be a mechanism by which prenatal BPA exposure affects behavior in young children. This study provides important new information about the potential effects of prenatal BPA exposure on human brain development and lays the foundation for future studies evaluating exposure at different times in pregnancy, as well as the effects of cumulative exposure across pregnancy and/or early childhood.

## Data Availability

The datasets used and/or analysed in the current study are available from the corresponding author on reasonable request.

## Abbreviations

APrON: Alberta Pregnancy Outcomes and Nutrition
BPA: Bisphenol A
CBCL: Child Behavior Checklist
DTI: Diffusion tensor imaging
ECDs: Endocrine-disrupting chemicals
FA: Fractional anisotropy
FDR: False discovery rate
LOD: Limit of Detection
MD: Mean diffusivity
MRI: Magnetic resonance imaging
ROI: Region of Interest
RSD: Relative standard deviation

## References

[1] Grandjean P, Landrigan PJ (2014). Neurobehavioural effects of developmental toxicity. Lancet Neurol.

[2] Vandenberg LN, Hauser R, Marcus M, Olea N, Welshons WV (2007). Human exposure to bisphenol a (BPA). Reprod Toxicol.

[3] Health Canada. Fourth report on human biomonitoring of environmental chemicals in Canada: results of the Canadian health measures survey cycle 4 (2014–2015); 2017. Retrieved October 3, 2018, from https://www.canada.ca/en/health-canada/services/environmental-workplace-health/reports-publications/environmental-contaminants/fourth-report-human-biomonitoring-environmental-chemicals-canada.html

[4] Braun JM, Yolton K, Dietrich KN, Hornung R, Ye X, Calafat AM, Lanphear BP (2009). Prenatal bisphenol a exposure and early childhood behavior. Environ Health Perspect.

[5] Braun JM, Kalkbrenner AE, Calafat AM, Yolton K, Ye X, Dietrich KN, Lanphear BP (2011). Impact of early-life bisphenol a exposure on behavior and executive function in children. Pediatrics.

[6] Atladottir HO, Gyllenberg D, Langridge A, Sandin S, Hansen SN, Leonard H (2015). The increasing prevalence of reported diagnoses of childhood psychiatric disorders: a descriptive multinational comparison. Eur Child Adolesc Psychiatry.

[7] Ejaredar M, Lee Y, Roberts DJ, Sauve R, Dewey D (2017). Bisphenol a exposure and children’s behavior: a systematic review. J Expo Sci Environ Epidemiol.

[8] World Health Organization (2010). Global plan of action for children’s health and the environment.

[9] Vandenberg LN, Chahoud I, Heindel JJ, Padmanabhan V, Paumgartten FJ, Schoenfelder G (2010). Urinary, circulating, and tissue biomonitoring studies indicate widespread exposure to bisphenol a. Environ Health Perspect.

[10] LaKind JS, Naiman DQ (2015). Temporal trends in bisphenol a exposure in the United States from 2003-2012 and factors associated with BPA exposure: spot samples and urine dilution complicate data interpretation. Environ Res.

[11] Schönfelder G, Wittfoht W, Hopp H, Talsness CE, Paul M, Chahoud I (2002). Parent bisphenol a accumulation in the human maternal-fetal-placental unit. Environ Health Perspect.

[12] Padmanabhan V, Siefert K, Ransom S, Johnson T, Pinkerton J, Anderson L (2008). Maternal bisphenol-a levels at delivery: a looming problem?. J Perinatol.

[13] Callan AC, Hinwood AL, Heffernan A, Eaglesham G, Mueller J, Odland JØ (2013). Urinary bisphenol a concentrations in pregnant women. Int J Hyg Environ Health.

[14] Balakrishnan B, Henare K, Thorstensen EB, Ponnampalam AP, Mitchell MD (2010). Transfer of bisphenol a across the human placenta. Am J Obstet Gynecol.

[15] Cheng CY, Wong EW, Lie PP, Li MW, Mruk DD, Yan HH (2011). Regulation of blood-testis barrier dynamics by desmosome, gap junction, hemidesmosome and polarity proteins: an unexpected turn of events. Spermatogenesis.

[16] Kubo K, Arai O, Omura M, Watanabe R, Ogata R, Aou S (2003). Low dose effects of bisphenol a on sexual differentiation of the brain and behavior in rats. Neurosci Res.

[17] Palanza P, Gioiosa L, Vom Saal FS, Parmigiani S (2008). Effects of developmental exposure to bisphenol a on brain and behavior in mice. Environ Res.

[18] Poimenova A, Markaki E, Rahiotis C, Kitraki E (2010). Corticosterone-regulated actions in the rat brain are affected by perinatal exposure to low dose of bisphenol a. Neuroscience.

[19] Chevrier J, Gunier RB, Bradman A, Holland NT, Calafat AM, Eskenazi B, Harley KG (2013). Maternal urinary bisphenol a during pregnancy and maternal and neonatal thyroid function in the CHAMACOS study. Environ Health Perspect.

[20] Corbel T, Perdu E, Gayrard V, Puel S, Lacroix MZ, Viguie C (2015). Conjugation and deconjugation reactions within the feto-placental compartment in a sheep model: a key factor determining bisphenol a fetal exposure. Drug Metab Dispos.

[21] Liu J, Li J, Wu Y, Zhao Y, Luo F, Li S (2017). Bisphenol a metabolites and bisphenol S in paired maternal and cord serum. Environ Sci Technol.

[22] Mendonca K, Hauser R, Calafat AM, Arbuckle TE, Duty SM (2014). Bisphenol a concentrations in maternal breast milk and infant urine. Int Arch Occup Environ Health.

[23] Kubo K, Arai O, Omura M, Watanabe R, Ogata R, Aou S (2003). Low dose effects of bisphenol a on sexual differentiation of the brain and behavior in rats. Neurosci Res.

[24] Palanza P, Gioiosa L, Vom Saal FS, Parmigiani S (2008). Effects of developmental exposure to bisphenol a on brain and behavior in mice. Environ Res.

[25] Poimenova A, Markaki E, Rahiotis C, Kitraki E (2010). Corticosterone-regulated actions in the rat brain are affected by perinatal exposure to low dose of bisphenol a. Neuroscience.

[26] Chevrier J, Gunier RB, Bradman A, Holland NT, Calafat AM, Eskenazi B, Harley KG (2013). Maternal urinary bisphenol a during pregnancy and maternal and neonatal thyroid function in the CHAMACOS study. Environ Health Perspect.

[27] Giesbrecht GF, Liu J, Ejaredar M, Dewey D, Letourneau N, Campbell T, Martin JW (2016). Urinary bisphenol a is associated with dysregulation of HPA-axis function in pregnant women: findings from the APrON cohort study. Environ Res.

[28] Ellahi M, ur Rashid M. The toxic effects BPA on fetuses, infants, and children. Bisphenol A exposure and health risks. 2017. 10.5772/intechopen.68896.

[29] Szymanska K, Gonkowski S (2018). Bisphenol A-induced changes in the enteric nervous system of the porcine duodenum. Neurotoxicology.

[30] Treit S, Lebel C, Baugh L, Rasmussen C, Andrew G, Beaulieu C (2013). Longitudinal MRI reveals altered trajectory of brain development during childhood and adolescence in fetal alcohol spectrum disorders. J Neurosci.

[31] Gautam P, Nuñez SC, Narr KL, Kan EC, Sowell ER (2014). Effects of prenatal alcohol exposure on the development of white matter volume and change in executive function. Neuroimage Clin.

[32] Jansson T, Powell TL (2007). Role of the placenta in fetal programming: underlying mechanisms and potential interventional approaches. Clin Sci.

[33] United States Environmental Protection Agency (EPA) (1988). Integrated risk information system (CASRN 80–05-7) oral RfD assessment: bisphenol A.

[34] Tian YH, Baek JH, Lee SY, Jang CG (2010). Prenatal and postnatal exposure to bisphenol a induces anxiolytic behaviors and cognitive deficits in mice. Synapse.

[35] Xu XH, Zhang J, Wang YM, Ye YP, Luo QQ (2010). Perinatal exposure to bisphenol-a impairs learning-memory by concomitant down-regulation of N-methyl-d-aspartate receptors of hippocampus in male offspring mice. Horm Behav.

[36] Xu X, Liu X, Zhang Q, Zhang G, Lu Y, Ruan Q (2013). Sex-specific effects of bisphenol-a on memory and synaptic structural modification in hippocampus of adult mice. Horm Behav.

[37] Farabollini F, Porrini S (1999). Dessì-Fulgheri F. perinatal exposure to the estrogenic pollutant bisphenol a affects behavior in male and female rats. Pharmacol Biochem Behav.

[38] Kubo K, Arai O, Ogata R, Omura M, Hori T, Aou S (2001). Exposure to bisphenol a during the fetal and suckling periods disrupts sexual differentiation of the locus coeruleus and of behavior in the rat. Neurosci Lett.

[39] Dessì-Fulgheri F, Porrini S, Farabollini F (2002). Effects of perinatal exposure to bisphenol a on play behavior of female and male juvenile rats. Environ Health Perspect.

[40] Farabollini F, Porrini S, Della SD, Bianchi F, Dessì-Fulgheri F (2002). Effects of perinatal exposure to bisphenol a on sociosexual behavior of female and male rats. Environ Health Perspect.

[41] Kawai K, Nozaki T, Nishikata H, Aou S, Takii M, Kubo C (2003). Aggressive behavior and serum testosterone concentration during the maturation process of male mice: the effects of fetal exposure to bisphenol a. Environ Health Perspect.

[42] Porrini S, Belloni V, Seta DD, Farabollini F, Giannelli G, Dessì-Fulgheri F (2005). Early exposure to a low dose of bisphenol a affects socio-sexual behavior of juvenile female rats. Brain Res Bull.

[43] Seta DD, Minder I, Dessì-Fulgheri F, Farabollini F (2005). Bisphenol-a exposure during pregnancy and lactation affects maternal behavior in rats. Brain Res Bull.

[44] Tiwari SK, Agarwal S, Chauhan LKS, Mishra VN, Chaturvedi RK (2015). Bisphenol-a impairs myelination potential during development in the hippocampus of the rat brain. Mol Neurobiol.

[45] Miodovnik A, Engel SM, Zhu C, Ye X, Soorya LV, Silva MJ (2011). Endocrine disruptors and childhood social impairment. Neurotoxicology.

[46] Yolton K, Xu Y, Strauss D, Altaye M, Calafat AM, Khoury J (2011). Prenatal exposure to bisphenol a and phthalates and infant neurobehavior. Neurotoxicol Teratol.

[47] Perera F, Vishnevetsky J, Herbstman JB, Calafat AM, Xiong W, Rauh V (2012). Prenatal bisphenol a exposure and child behavior in an inner-city cohort. Environ Health Perspect.

[48] Harley KG, Gunier RG, Kogut K, Johnson C, Bradman A, Calafat AM (2013). Prenatal and early childhood bisphenol a concentrations and behavior in school-aged children. Environ Res.

[49] Hong SB, Hong YC, Kim JW, Park EJ, Shin MS, Kim BN (2013). Bisphenol a in relation to behavior and learning of school-age children. J Child Psychol Psychiatry.

[50] Braun JM, Kalkbrenner AE, Just AC, Yolton K, Calafat AM, Sjödin A (2014). Gestational exposure to endocrine-disrupting chemicals and reciprocal social, repetitive, and stereotypic behaviors in 4- and 5-year-old children: the HOME study. Environ Health Perspect.

[51] Evans SF, Kobrosly RW, Barrett ES, Thurston SW, Calafat AM, Weiss B (2014). Prenatal bisphenol a exposure and maternally reported behavior in boys and girls. Neurotoxicology.

[52] Roen EL, Wang Y, Calafat AM, Wang S, Margolis A, Herbstman J (2015). Bisphenol a exposure and behavioral problems among inner city children at 7–9 years of age. Environ Res.

[53] Stein TP, Schluter MD, Steer RA, Guo L, Ming X (2015). Bisphenol a exposure in children with autism spectrum disorders. Autism Res.

[54] Perera F, Nolte ELR, Wang Y, Margolis AE, Calafat AM, Wang S (2016). Bisphenol a exposure and symptoms of anxiety and depression among inner city children at 10–12 years of age. Environ Res.

[55] Lenroot RK, Giedd JN (2006). Brain development in children and adolescents: insights from anatomical magnetic resonance imaging. Neurosci Biobehav Rev.

[56] Albaugh MD, Ducharme S, Karama S, Watts R, Lewis JD, Orr C (2017). Anxious/depressed symptoms are related to microstructural maturation of white matter in typically developing youths. Dev Psychopathol.

[57] Bahn GH, Hong M, Lee KM, Lee C, Ryu CW, Lee JA (2018). The relationship between microstructural alterations of the brain and clinical measurements in children and adolescents with hair pulling disorder. Brain Imaging Behav.

[58] Loe IM, Lee ES, Feldman HM (2013). Attention and internalizing behaviors in relation to white matter in children born preterm. J Dev Behav Pediatr.

[59] de Zeeuw P, Mandl RC, Hulshoff Pol HE, van Engeland H, Durston S (2012). Decreased frontostriatal microstructural organization in attention deficit/hyperactivity disorder. Hum Brain Mapp.

[60] Achenbach TM, Rescorla LA (2001). Manual for the ASEBA school-age forms & profiles.

[61] Kaplan BJ, Giesbrecht GF, Leung BMY, Field CJ, Dewey D, Bell RC (2014). The Alberta pregnancy outcomes and nutrition (APrON) cohort study: rationale and methods. Matern Child Nutr.

[62] Liu J, Wattar N, Field CJ, Dinu I, Dewey D, Martin JW (2018). Exposure and dietary sources of bisphenol a (BPA) and BPA-alternatives among mothers in the APrON cohort study. Environ Int.

[63] Thieba C, Frayne A, Walton M, Mah A, Benischek A, Dewey D (2018). Factors associated with successful MRI scanning in unsedated young children. Front Pediatr.

[64] Leemans AJ, Jeurissen B, Sijbers J, Jones DK (2009). ExploreDTI: A graphical toolbox for processing, analyzing, and visualizing diffusion MR data. Proc Int Soc Mag Reson Med.

[65] Wakana S, Jiang H, Nagae-Poetscher LM, van Zijl PCM, Mori S (2004). Fiber tract–based atlas of human white matter anatomy. Radiology.

[66] Reynolds J. Grohs M. Lebel, C. White matter tractography guides. Figsare. 2019. Retrieved from https://figshare.com/articles/White _Matter_Tractography_Guides/7603271/1

[67] Beaulieu C (2002). The basis of anisotropic water diffusion in the nervous system - a technical review. NMR Biomed.

[68] Qiu A, Mori S, Miller MI (2015). Diffusion tensor imaging for understanding brain development in early life. Annu Rev Psychol.

[69] Lebel C, Deoni S (2018). The development of brain white matter microstructure. Neuroimage.

[70] Warnick EM, Bracken MB, Kasl S (2008). Screening efficiency of the child behavior checklist and strengths and difficulties questionnaire: a systematic review. Child Adoles Ment Health.

[71] Perera F, Vishnevetsky J, Herbstman JB, Calafat AM, Xiong W, Rauh V (2012). Prenatal bisphenol a exposure and child behavior in an inner-city cohort. Environ Health Perspect.

[72] Roen EL, WangY CAM, Wang S, Margolis A, Herbstman J (2015). Bisphenol a exposure and behavioral problems among inner city children at 7-9 years of age. Environ Res.

[73] Sterba SK, Prinstein MJ, Cox MJ (2007). Trajectories of internalizing problems across childhood: heterogeneity, external validity, and gender differences. Dev Psychopathol.

[74] Miettunen J, Murray GK, Jones PB, Mäki P, Ebeling H, Taanila A (2014). Longitudinal associations between childhood and adulthood externalizing and internalizing psychopathology and adolescent substance use. Psychol Med.

[75] Hayes AF (2017). Introduction to mediciation, moderation, and conditional process analysis: a regression-based approach.

[76] Fairchild AJ, MacKinnon DP (2009). A general model for testing mediation and moderation effects. Prev Sci.

[77] Baron RM, Kenny DA (1986). The moderator-mediator variable distinction in social psychological research: conceptual, strategic, and statistical considerations. J Pers Soc Psychol.

[78] Zhao X, Lynch JG, Chen Q (2010). Reconsidering Baron and Kenny: myths and truths about mediation analysis. J Consum Res.

[79] Shrout PE, Bolger N (2002). Mediation in experimental and nonexperimental studies: new procedures and recommendations. Psychol Methods.

[80] Lakind JS, Levesque J, Dumas P, Bryan S, Clarke J, Naiman DQ (2012). Comparing United States and Canadian population exposures from National Biomonitoring Surveys: bisphenol a intake as a case study. J Expo Sci Environ Epidemiol.

[81] Kinch CD, Ibhazehiebo K, Jeong JH, Habibi HR, Kurrasch DM (2015). Low-dose exposure to bisphenol a and replacement bisphenol S induces precocious hypothalamic neurogenesis in embryonic zebrafish. Proc Natl Acad Sci U S A.

[82] Iwakura T, Iwafuchi M, Muraoka D, Yokosuka M, Shiga T, Watanabe C (2010). In vitro effects of bisphenol a on developing hypothalamic neurons. Toxicology.

[83] Kundakovic M, Champagne FA (2011). Epigenetic perspective on the developmental effects of bisphenol a. Brain Behav Immun.

[84] Wolstenholme JT, Rissman EF, Connelly JJ (2011). The role of bisphenol a in shaping the brain, epigenome and behavior. Horm Behav.

[85] Lebel C, Treit S, Beaulieu C (2019). A review of diffusion MRI of typical white matter development from early childhood to young adulthood. NMR Biomed.

[86] Scholz J, Tomassini V, Johansen-Berg H, Johansen-Berg H, Behrens TEJ (2014). Individual differences in white matter microstructure in the healthy brain. Diffusion MRI: from quantitative measurement to in vivo neuroanatomy.

[87] Nagel BJ, Bathula D, Herting M, Schmitt C, Kroenke CD, Fair D (2011). Altered white matter microstructure in children with attention-deficit/hyperactivity disorder. J Am Acad Child Adolesc Psychiatry.

[88] Loe IM, Lee ES, Feldman HM (2013). Attention and internalizing behaviors in relation to white matter in children born preterm. J Dev Behavioral Pediatr.

[89] Aghajani M, Veer IM, van Lang ND, Meens PH, van den Bulk BG, Rombouts SA (2014). Altered white-matter architecture in treatment-naive adolescents with clinical depression. Psychol Med.

[90] Ortibus E, Verhoeven J, Sunaert S, Casteels I, de Cock P, Lagae L (2012). Integrity of the inferior longitudinal fasciculus and impaired object recognition in children: a diffusion tensor imaging study. Dev Med Child Neurol.

[91] Knyazeva MG. Splenium of corpus callosum: patterns of interhemispheric interaction in children and adults. Neural Plast. 2013;639430. 10.1155/2013/639430 PMC3610378 23577273

[92] Meeker JD (2012). Exposure to environmental endocrine disruptors and child development. Arch Pediatr Adolesc Med.

[93] Bellinger DC (2013). Prenatal exposures to environmental chemicals and children’s neurodevelopment: an update. Saf Health Work.

[94] Mesman J, Bongers IL, Koot HM (2001). Preschool developmental pathways to preadolescent internalizing and externalizing problems. J Child Psychol Psychiatry.

[95] Huang H, Xue R, Zhang J, Ren T, Richards LJ, Yarowsky P (2009). Anatomical characterization of human fetal brain development with diffusion tensor magnetic resonance imaging. J Neurosci.

[96] Dubois J, Dehaene-Lambertz G, Kulikova S, Poupon C, Hüppi PS, Hertz-Pannier L (2014). The early development of brain white matter: a review of imaging studies in fetuses, newborns and infants. Neuroscience.

[97] Lebel C, Beaulieu C (2011). Longitudinal development of human brain wiring continues from childhood into adulthood. J Neurosci.

[98] Sampaio RC, Truwit CL, Collins ML, Nelson CA (2001). Myelination in the developing human brain. Handbook of developmental cognitive neuroscience.

[99] Rodier PM (1995). Developing brain as a target of toxicity. Environ Health Perspect.

[100] Miodovnik A (2011). Environmental neurotoxicants and developing brain. Mt Sinai J Med.

[101] Negri-Cesi P (2015). Bisphenol a interaction with brain development and functions. Dose Response.

[102] Emery B (2010). Regulation of oligodendrocyte differentiation and myelination. Science.

[103] Budday S, Steinmann P, Kuhl E (2015). Physical biology of human brain development. Front Cell Neurosci.

[104] Mallozzi M, Bordi G, Garo C, Caserta D (2016). The effect of maternal exposure to endocrine disrupting chemicals on fetal and neonatal development: a review on the major concerns. Birth Defects Res C Embryo Today.

[105] Sandman CA, Davis EP, Buss C, Glynn LM (2012). Exposure to prenatal psychobiological stress exerts programming influences on the mother and her fetus. Neuroendocrinology.

[106] Jansson T, Powell TL (2007). Role of the placenta in fetal programming: underlying mechanisms and potential interventional approaches. Clinical Sci (Lond).

[107] Mahalingaiah S, Meeker JD, Pearson KR, Calafat AM, Ye X, Petrozza J (2008). Temporal variability and predictors of urinary bisphenol a concentrations in men and women. Environ Health Perspect.

[108] Ye X, Wong LY, Bishop AM, Calafat AM (2011). Variability of urinary concentrations of bisphenol a in spot samples, first morning voids, and 24-hour collections. Environ Health Perspect.

[109] Perrier F, Giorgis-Allemand L, Slama R, Philippat C (2016). Within-subject pooling of biological samples to reduce exposure misclassification in biomarker-based studies. Epidemiology.

[110] Dean DC, O’Muircheartaigh J, Dirks H, Waskiewicz N, Walker L, Doernberg E (2015). Characterizing longitudinal white matter development during early childhood. Brain Struct Funct.

[111] Reynolds JE, Grohs MN, Dewey D, Lebel C (2019). Global and regional white matter development in early childhood. Neuroimage.

